# Stereotactic Microwave Ablation of Early-Stage Hepatocellular Carcinoma in Patients with Transjugular Intrahepatic Portosystemic Shunts: A Matched Case–Control Study

**DOI:** 10.1007/s00270-026-04522-3

**Published:** 2026-07-03

**Authors:** Liang Zhang, Quirin D. Strotzer, Laura S. Kupke, Vinzenz Mayr, Georg H. Hiernickel, Moritz Brandenstein, Lukas Luerken, Simon Jage, Gregor Scharf, Christian Stroszczynski, Ingo Einspieler

**Affiliations:** Department of Radiology, University Hospital Regensburg, Franz-Josef-Strauß-Allee 11, 93053 Regensburg, Germany

**Keywords:** Hepatocellular carcinoma, Microwave ablation, Transjugular intrahepatic portosystemic shunt, Stereotactic navigation, Liver cirrhosis, Thermal ablation

## Abstract

**Purpose:**

To assess safety and outcomes of stereotactic CT-guided microwave ablation (MWA) for hepatocellular carcinoma (HCC) in patients with transjugular intrahepatic portosystemic shunts (TIPS) versus a matched control group without TIPS.

**Materials and Methods:**

Retrospective matched case–control study of cirrhotic patients with HCC treated with stereotactic CT-guided MWA. TIPS patients were matched 1:1 to controls using hierarchical matching based on Child–Pugh class, number of treated tumors, MELD score, tumor diameter, and ablation date. Primary endpoints were primary technique efficacy (PTE), local tumor progression (LTP), 30-day complications, and 30-day all-cause mortality. Secondary endpoints were 12-month Kaplan–Meier estimates for progression-free survival (PFS), hepatic decompensation-free survival (HDFS), and overall survival (OS), compared using log-rank tests.

**Results:**

46 patients (23 TIPS, 23 controls) with 62 index lesions were analyzed. PTE was achieved in 30/31 lesions (96.8%) in both groups. Among lesions with PTE, LTP occurred in 1/30 lesions (3.3%) in the TIPS group and 0/30 lesions (0%) in controls. Within 30 days, two major complications occurred in the TIPS group and one in the control group; no 30-day deaths occurred. Observed 12-month estimates did not differ significantly between groups for PFS (52.4% vs. 46.0%, *p* = 0.876), HDFS (71.8% vs. 76.9%, *p* = 0.519), or OS (91.1% vs. 90.0%, *p* = 0.903). Child–Pugh A was associated with higher HDFS than Child–Pugh B (95.8% vs. 50.2%, *p* = 0.001).

**Conclusion:**

Stereotactic CT-guided MWA of early-stage HCC appears feasible in carefully selected patients with TIPS, warranting further evaluation in larger cohorts.

**Level of Evidence:**

3b.

**Graphical abstract:**

**Figure:**
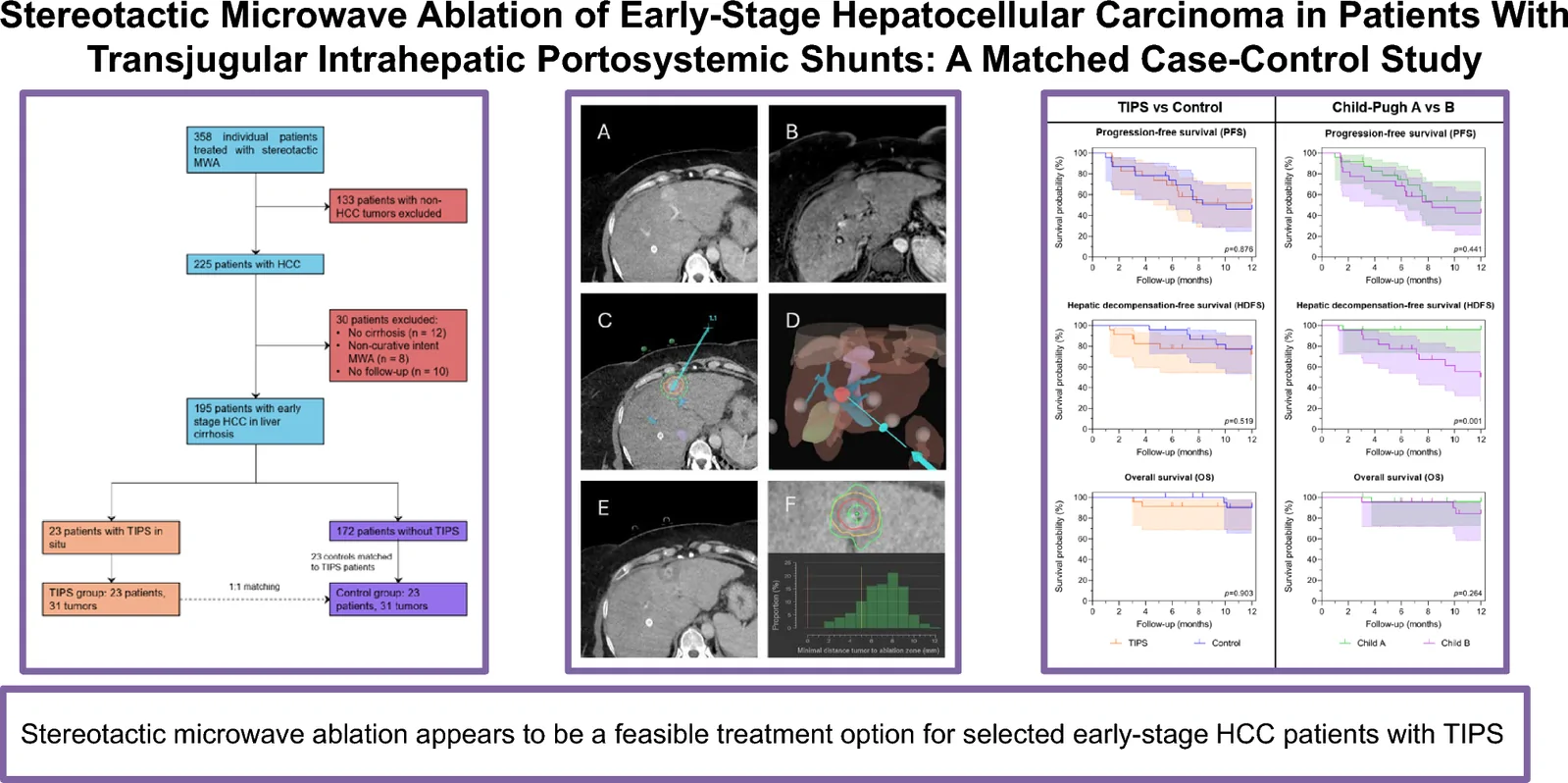

## Introduction

Hepatocellular carcinoma (HCC) is the most common primary liver malignancy and a leading cause of cancer-related mortality worldwide, with most cases arising in patients with cirrhosis [1–3]. While thermal ablation, including microwave ablation (MWA), is a standard-of-care option for early-stage disease [1, 4–7], a transjugular intrahepatic portosystemic shunt (TIPS) may modify the physiologic setting in which thermal ablation is performed. As TIPS placement and thermal ablation for HCC are increasingly encountered in the same cirrhotic population [8, 9], it is important to define how shunted hemodynamics influence the efficacy and safety of MWA.

By creating a low-resistance portosystemic conduit, TIPS diverts portal venous flow and reshapes intrahepatic perfusion [10, 11]. This altered hemodynamic environment may also promote relative hepatic arterialization, which has been discussed as a potential contributor to HCC development in cirrhotic patients. For thermal ablation, these perfusion changes raise two related questions. First, altered perfusion may change convective heat loss and reduce the predictability of thermal deposition and margin formation, potentially compromising local tumor control even when tumors are not directly adjacent to the shunt [12–14]. Second, procedural safety is a concern, including bleeding during needle placement and whether peri-ablation effects could impair shunt patency [15, 16]. In addition, because TIPS recipients often have limited hepatic reserve and altered hepatic hemodynamics, even modest parenchymal injury from MWA may precipitate hepatic decompensation, which can compete with tumor progression and influence survival and post-ablation oncologic course [17–21].

While evidence for the safety of radiofrequency ablation exists for patients with TIPS, data for MWA remain limited and comparative analyses with well-matched controls are scarce [22–25]. The study by Dumoutier et al. is the closest prior comparative analysis in this setting, but non-TIPS controls were selected consecutively according to treatment timing, and treatment included both radiofrequency and microwave ablation, with radiofrequency ablation used in most cases. To address these gaps, the present analysis focused exclusively on stereotactic CT-guided microwave ablation and selected controls according to predefined variables reflecting hepatic reserve and tumor burden.

## Materials and Methods

### Patients and Study Design

This single-center retrospective matched case–control study was approved by the local ethics committee, informed consent was waived. Consecutive patients undergoing stereotactic MWA between January 2020 and February 2026 were screened. Exclusions were non-HCC tumors, absence of cirrhosis, tumor burden unsuitable for curative-intent ablation (more than three lesions or any lesion greater than 3 cm), or missing follow-up imaging. After applying the exclusion criteria, the eligible study cohort comprised adults with cirrhosis and HCC treated with curative intent in an early-stage setting. The index ablation was defined as the patient’s first stereotactic MWA within the study period.

All consecutive patients with a TIPS in situ at index ablation formed the TIPS group. Patients without TIPS from the same screened cohort formed the control pool. Controls were selected using a predefined hierarchical 1:1 nearest-neighbor matching strategy. Exact matching was performed for Child–Pugh class and number of treated tumors. Among eligible controls, nearest-neighbor selection prioritized MELD score, with preference for a difference of ≤ 3 points, followed by maximum tumor diameter within ± 5 mm whenever possible and closest ablation date. In patients with multiple treated tumors, similarity of total tumor diameter was used as an additional tie-breaker.

## Stereotactic Microwave Ablation and Follow-Up

**Fig. 1:**
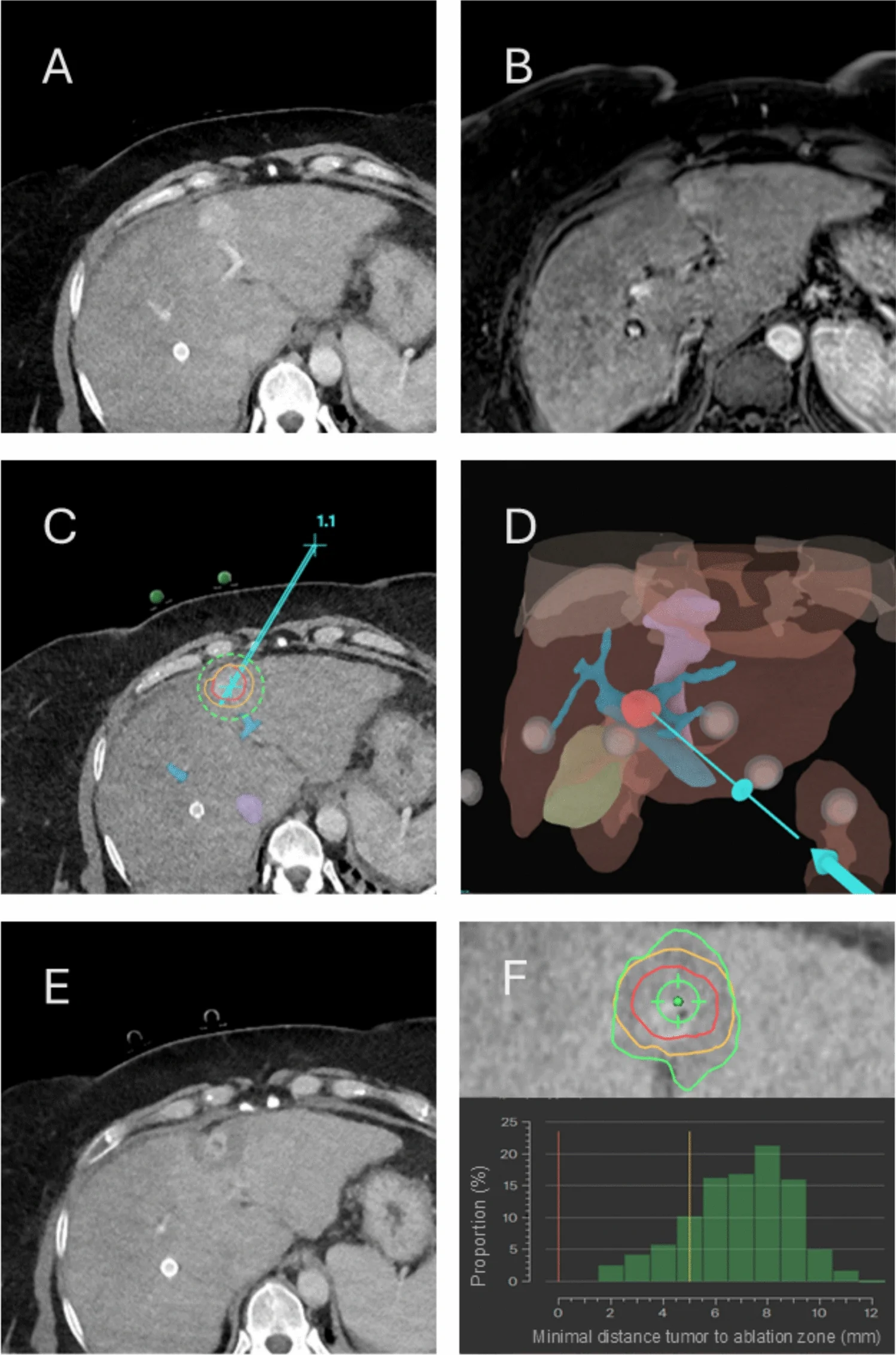
Illustrative workflow of stereotactic CT-guided microwave ablation for a subcapsular HCC in the left hepatic lobe in a patient with TIPS. **A**–**B** Pre-procedural arterial phase CT and MRI demonstrating the subcapsular left-lobe HCC. **C** Stereotactic planning with trajectory and safety margins. **D** Three-dimensional planning view illustrating the relationship between the tumor, planned needle path, vascular structures, and external radiopaque fiducials used for stereotactic registration. **E** Immediate post-ablation CT demonstrating the ablation zone covering the tumor. **F** Software-based margin confirmation (AblaSure, CAScination AG, Bern, Switzerland) after image co-registration of pre- and post-ablation CT. The segmented tumor is shown in red, the 5 mm safety margin in yellow, and the ablation zone in green. The histogram displays the distribution of minimal distances from the tumor surface to the ablation-zone boundary; values to the right of 0 mm indicate tumor coverage, and the red and yellow vertical markers indicate the 0-mm tumor boundary and the planned 5-mm ablative margin, respectively

Treatment indications for stereotactic microwave ablation were confirmed in an interdisciplinary tumor board. Under general anesthesia, stereotactic MWA was performed in an interventional CT suite with respiratory motion control using end-expiratory breath-holds or high-frequency jet ventilation (illustrative case shown in Fig. 1). All CT images were acquired on a 128-slice CT scanner (SOMATOM Definition Edge, Siemens Healthineers, Erlangen, Germany). Patients were positioned supine or slightly right-elevated and immobilized on a vacuum mattress. After sterile preparation, radiopaque skin fiducials were applied for stereotactic registration. A biphasic contrast-enhanced planning CT (arterial and portal venous phases) was acquired under motion control after intravenous administration of 100 mL iohexol (Accupaque™ 350; GE Healthcare Buchler GmbH & Co. KG, Braunschweig, Germany).

Planning CT data were transferred to a stereotactic navigation system (CAS-One IR, CAScination AG, Bern, Switzerland) for trajectory planning to ensure complete target coverage while avoiding critical structures. Using the stereotactic aiming device, the microwave antenna was advanced percutaneously under navigation guidance, with position confirmation on unenhanced CT and iterative adjustment as needed. Microwave ablation was performed using one of three commercially available platforms (Dophi™ M150E, Surgnova Healthcare Technologies Co., Ltd., Beijing, China; Emprint™ HP, Medtronic, Minneapolis, MN, USA; or NEUWAVE™ PRXT, NeuWave Medical, Inc., Madison, WI, USA) depending on availability and operator preference. Power and application time were selected in a lesion-size-adapted manner using system-specific reference tables stored in the navigation software, targeting complete tumor coverage with an intended circumferential ablative margin of at least 5 mm. Track ablation was performed during antenna withdrawal, followed by immediate biphasic contrast-enhanced CT to assess early complications and confirm ablation coverage. Ablation confirmation was performed using dedicated three-dimensional ablation confirmation software (AblaSure, CAScination AG, Bern, Switzerland) integrated within the CAS-One IR platform. The software enables quantitative assessment of the achieved ablative margin relative to the segmented tumor and planned circumferential target margin.

Follow-up was performed with multiphase contrast-enhanced liver MRI using a hepatocyte-specific contrast agent (Primovist) at 6 weeks, 3 months, and every 3 months thereafter on a 3 T system (MAGNETOM Skyra, Siemens Healthineers); dual-phase contrast-enhanced CT was used when MRI was not feasible. Routine abdominal ultrasound was performed at the same intervals in all patients, including Doppler assessment of TIPS patency in the TIPS group.

## Endpoints

Primary endpoints were primary technique efficacy (PTE, lesion level), local tumor progression (LTP, lesion level), 30-day complications, and 30-day mortality. PTE was defined as complete tumor ablation without residual viable tumor on the first post-ablation contrast-enhanced follow-up imaging, routinely performed approximately 6 weeks after ablation. LTP was defined as the appearance of new viable tumor at or immediately adjacent to the ablation zone in a lesion that had previously achieved PTE. Complications were defined as any adverse event occurring within 30 days after MWA, including hepatic decompensation, and were graded according to the modified CIRSE classification system [26]. Hepatic decompensation was defined as a clinically evident event, including new onset or worsening of ascites, hepatic encephalopathy, or variceal bleeding. Stable pre-existing ascites was not counted as decompensation. Worsening ascites required a clear increase compared with pre-ablation imaging and/or treatment escalation, including new or intensified diuretics, therapeutic paracentesis, or hospitalization. Hepatic encephalopathy and variceal bleeding were adjudicated from clinical documentation and treatment requirement.

Secondary endpoints were 12-month Kaplan–Meier estimates for progression-free survival (PFS), hepatic decompensation-free survival (HDFS), and overall survival (OS). PFS was defined as time from ablation to first tumor progression (LTP, intrahepatic distant recurrence, or extrahepatic progression) or death from any cause; HDFS as time to first hepatic decompensation or death from any cause; and OS as time to death from any cause.

## Statistical Analysis

Analyses were performed using IBM SPSS Statistics (version 28.0). Continuous variables are summarized as median (interquartile range) and categorical variables as n/N (%). Between-group comparisons (TIPS vs. control group) were performed using the Mann–Whitney U test for continuous variables, Fisher exact test for categorical variables, and the chi-square test for multi-category distributions. Time-to-event outcomes were analyzed using the Kaplan–Meier method, with group comparisons by the log-rank test (TIPS vs. control group). Additional Kaplan–Meier and log-rank analyses were performed stratified by Child–Pugh class (A vs. B). Univariable Cox proportional hazards regression was used to explore associations between key prognostic factors and PFS, OS, and HDFS, reporting hazard ratios with 95% confidence intervals; due to the limited number of events, these analyses were considered exploratory. *P*-values < 0.05 were considered statistically significant.

## Results

### Patient Characteristics

**Fig. 2:**
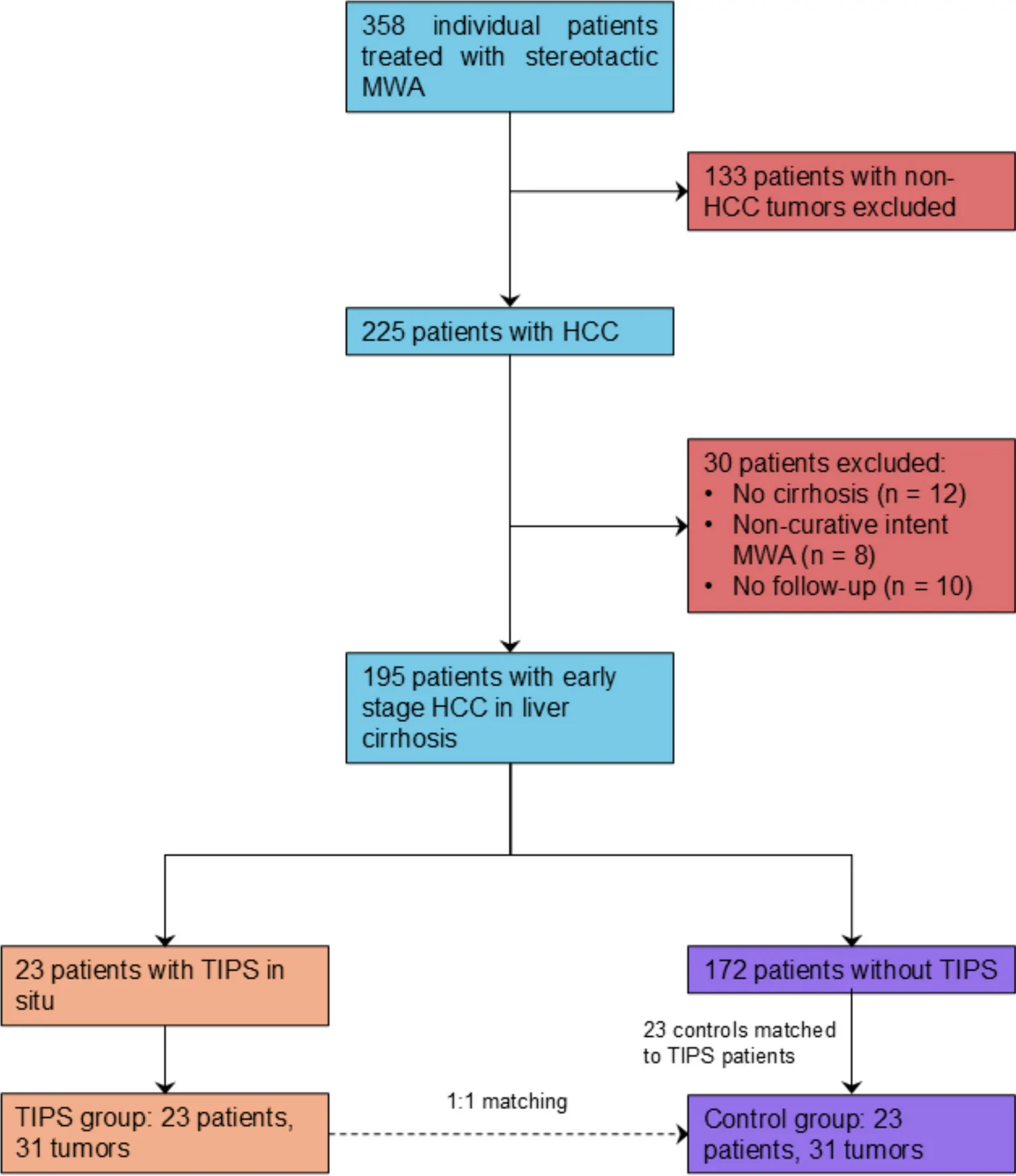
Flowchart of cohort selection and matching (TIPS vs. control group)

A total of 46 patients were included (23 with TIPS and 23 patients in the matched control group), and 62 index HCC lesions were treated overall (31 per group). The patient selection and matching process is summarized in the study flow chart (Fig. 2). Baseline characteristics of both groups are summarized in Table 1. No patient received systemic HCC therapy before index ablation. During follow-up, systemic therapy was initiated in 4 patients because of subsequent tumor progression.

**Table 1:** Baseline characteristics, tumor burden, lesion location distribution and procedural metrics

Variable	TIPS (n = 23)	Control (n = 23)	*p *value
*Demographics*			
Patients, n	23	23	
Age, years	64.0 (60.5–68.0)	67.0 (61.5–70.0)	0.311
Male sex	20/23 (87.0%)	19/23 (82.6%)	1.000
*Underlying liver disease and liver function*			
Alcohol-related liver disease	15/23 (65.2%)	12/23 (52.2%)	0.550
Child–Pugh class A	12/23 (52.2%)	12/23 (52.2%)	1.000
Child–Pugh class B	11/23 (47.8%)	11/23 (47.8%)	1.000
Model for end-stage liver disease (MELD) score	13.0 (10.5–15.0)	12.0 (9.5–14.5)	0.426
Ascites on pre-procedural imaging	6/23 (26.1%)	4/23 (17.4%)	0.722
*Tumor stage and biomarkers*			
BCLC stage at diagnosis			0.711
BCLC stage 0	8/23 (34.8%)	8/23 (34.8%)	
BCLC stage A	12/23 (52.2%)	10/23 (43.5%)	
BCLC stage B	3/23 (13.0%)	5/23 (21.7%)	
Prior HCC therapy	7/23 (30.4%)	9/23 (39.1%)	0.758
TACE	6/23 (26.1%)	6/23 (26.1%)	
Resection	1/23 (4.3%)	3/23 (13.0%)	
Thermal ablation	1/23 (4.3%)	0/23 (0.0%)	
AFP positive (> 7 ng/mL)	10/23 (43.5%)	7/23 (30.4%)	0.542
*Tumor burden at index ablation*			
Treated index lesions, n	31	31	1.000
1 index lesion	17/23 (73.9%)	17/23 (73.9%)	
2 index lesions	4/23 (17.4%)	4/23 (17.4%)	
3 index lesions	2/23 (8.7%)	2/23 (8.7%)	
Maximum tumor diameter, mm	17.0 (14.0–21.0)	17.0 (12.0–19.5)	0.421
*Tumor location features (lesion-level)*			
Subcapsular	15/31 (48.4%)	16/31 (51.6%)	1.000
Subdiaphragmatic or subcardiac	4/31 (12.9%)	7/31 (22.6%)	0.508
Perivascular (portal-hepatic vein adjacency)	4/31 (12.9%)	4/31 (12.9%)	1.000
*Procedural metrics*			
Procedure duration, min	73.0 (52.0–91.0)	69.0 (56.5–95.0)	0.775
Dose-length product (DLP), mGy cm	1989 (1636–2404)	1925 (1535–2141)	0.517
Ablation zone size (long axis), mm	36.0 (33.0–38.0)	36.0 (31.0–38.5)	0.534
Ablation zone size (short axis), mm	28.0 (21.5–32.0)	27.0 (24.0–31.0)	0.827
Minimum ablative margin, mm	5.0 (2.0–7.0)	5.0 (3.0–7.0)	0.846
Complete 5-mm margin achieved	19/31 (61.3%)	22/31 (71.0%)	0.592
MWA antenna model			0.831
Dophi	13/23 (56.5%)	14/23 (60.9%)	
Emprint HP	2/23 (8.7%)	1/23 (4.3%)	
NEUWAVE PRXT	8/23 (34.8%)	8/23 (34.8%)	

Data are presented as median (interquartile range) or n/N (%). *P*-values were calculated using the Mann–Whitney U test for continuous variables, Fisher exact test for categorical variables, and the chi-square test for multi-category distributions. Subcapsular, subdiaphragmatic, and subcardiac locations were defined as a tumor margin within 10 mm of the liver capsule, diaphragm, or cardiac border, respectively; perivascular location was defined as a tumor margin within 5 mm of a portal or hepatic venous branch. Complete 5-mm margin achieved was defined as a minimum ablative margin >  = 5 mm. HCC, hepatocellular carcinoma; TIPS, transjugular intrahepatic portosystemic shunt; BCLC, Barcelona Clinic Liver Cancer; AFP, alpha-fetoprotein; TACE, transarterial chemoembolization; MWA, microwave ablation

Median follow-up was 377 days (IQR 247-598) in the TIPS group and 427 days (IQR 340-648) in the control group. In the TIPS group, all shunts were patent on pre-procedural imaging before ablation, and no TIPS stent thrombosis or occlusion was observed during follow-up. Three patients underwent TIPS revision with percutaneous transluminal angioplasty after post-ablation hepatic decompensation with ascites. TIPS characteristics are provided in Table 2.

**Table 2:** TIPS characteristics

Variable	TIPS (n = 23)
*TIPS indication*	
Bleeding-related	12/23 (52.2%)
Refractory ascites	10/23 (43.5%)
Refractory hepatic hydrothorax	1/23 (4.3%)
*TIPS configuration and portal pressure gradient*	
Right hepatic vein to right portal vein	22/23 (95.7%)
Middle hepatic vein to left portal vein	1/23 (4.3%)
Pre-TIPS portal pressure gradient, mmHg	16 (14–17)
Post-TIPS portal pressure gradient, mmHg	6 (4–8)
*TIPS surveillance and revision*	
Prior TIPS revision	4/23 (17.4%)
TIPS revision after MWA	3/23 (13.0%)
TIPS flow velocity, cm/s	154 (132–165)
TIPS thrombosis or occlusion during follow-up	0/23 (0.0%)
*Tumor and ablation-zone relationship to TIPS*	
Minimum tumor-to-TIPS stent distance, mm	43 (34–61)
Minimum ablation-zone-to-TIPS stent distance, mm	34 (25–50)
Tumors within 20 mm of TIPS stent	4/31 (12.9%)
Ablation zones within 20 mm of TIPS stent	6/31 (19.4%)
*Pre-procedural Doppler hemodynamic assessment*	
Portal flow direction in the lesion-bearing liver lobe: hepatopetal	13/31 (41.9%)
Portal flow direction in the lesion-bearing liver lobe: hepatofugal	18/31 (58.1%)
Portal venous flow velocity, cm/s	27 (22–32)
Hepatic arterial peak systolic velocity, cm/s	122 (92–146)
Hepatic arterial resistive index	0.63 (0.58–0.65)

Data are presented as median (interquartile range) or n/N (%). Minimum tumor-to-TIPS stent distance was defined as the shortest distance from the tumor margin to the nearest outer border of the TIPS stent on pre-procedural cross-sectional imaging. Minimum ablation-zone-to-TIPS stent distance was defined as the shortest distance from the post-ablation zone margin to the nearest outer border of the TIPS stent. TIPS indication, configuration, pressure gradients, revision status, and Doppler velocity parameters are reported at the patient level. Tumor-to-TIPS proximity, ablation-zone-to-TIPS proximity, and portal flow direction in the lesion-bearing liver lobe are reported at the lesion level where applicable. TIPS, transjugular intrahepatic portosystemic shunt

## Primary Endpoints

### Primary Technique Efficacy

**Fig. 3:**
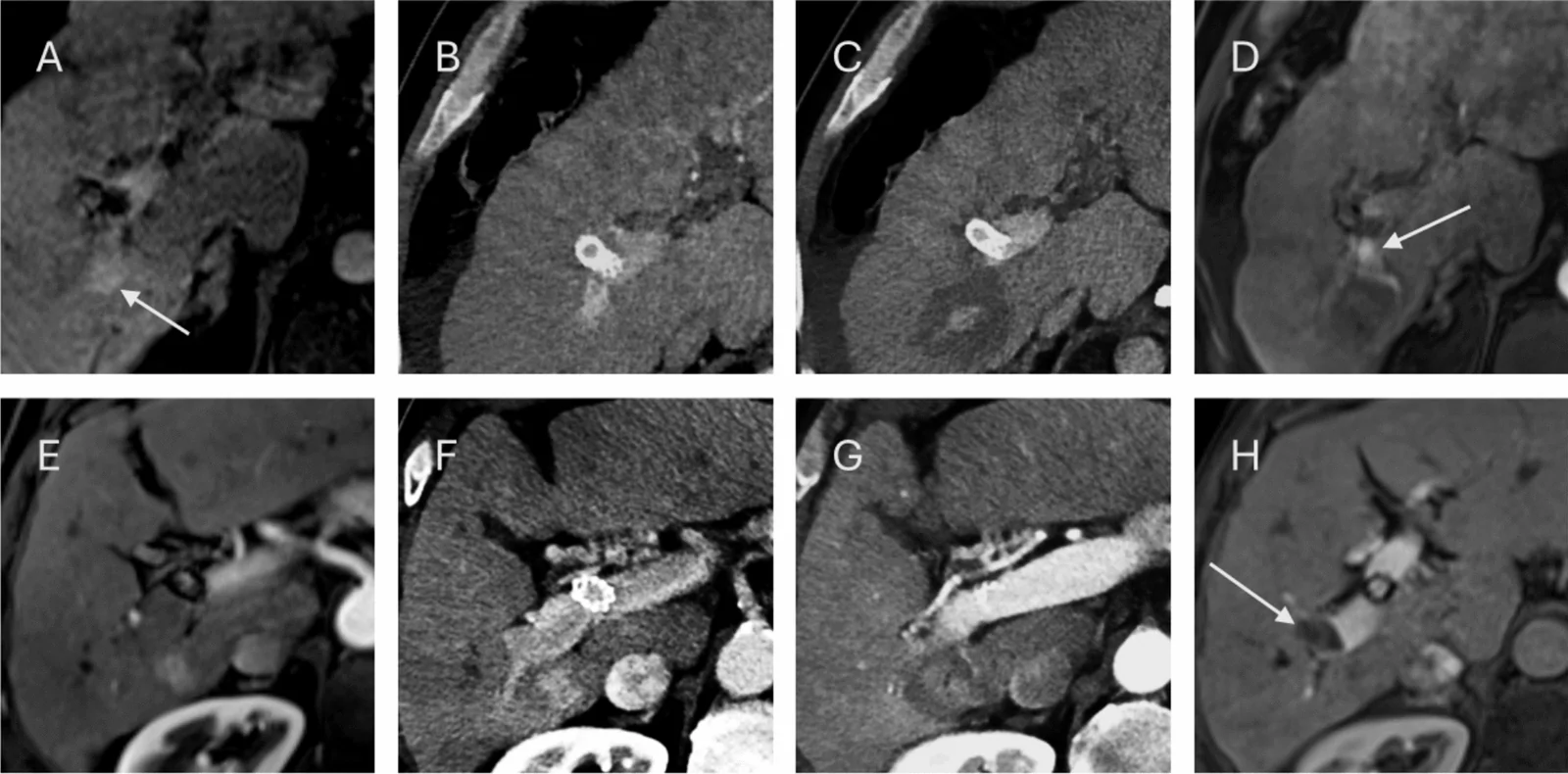
Examples of TIPS-related challenges during stereotactic microwave ablation. **A**–**D** Patient with HCC abutting the TIPS stent. Pre-procedural contrast-enhanced MRI shows the arterially enhancing HCC in contact with the shunt (arrow, **A**). Intraprocedural CT confirms minimal tumor-stent separation **B**. Immediate post-ablation CT shows the ablation zone abutting the stent, with minimal stent-facing margin **C**. First follow-up contrast-enhanced MRI shows residual viable tumor at the tumor-shunt interface (arrow, **D**), consistent with lack of primary technique efficacy. This case illustrates a setting in which margin formation may be technically challenging near the TIPS stent. **E**–**H** Patient with HCC adjacent to the right portal venous branch and TIPS inflow region. Pre-procedural contrast-enhanced MRI shows the arterially enhancing HCC near the right portal venous anatomy (**E**). Intraprocedural CT shows the TIPS stent and adjacent portal venous structures near the ablation trajectory (**F**). Immediate post-ablation CT shows the ablation zone extending along the right portal venous branch near the TIPS inflow region (**G**). Follow-up contrast-enhanced MRI shows partial right portal vein thrombosis (arrow, **H**). This case illustrates a setting in which ablation near the TIPS inflow region may be associated with periportal vascular injury

PTE was achieved in 30/31 lesions (96.8%) in both the TIPS group and the control group. The two lesions without PTE occurred in anatomically challenging settings, including one TIPS-group lesion near the shunt and one control-group lesion in a subdiaphragmatic location; both showed residual marginal tumor on first follow-up imaging. Representative TIPS-related challenging scenarios, including tumor contact with the shunt and periportal vascular change near the TIPS inflow region, are shown in Fig. 3.

### Local Tumor Progression

Among lesions with PTE (30/31 per group), LTP occurred in 1/30 lesions (3.3%) in the TIPS group and 0/30 (0%) in the control group.

### 30-Day Complications and Mortality

Within 30 days, two major complications occurred in the TIPS group (2/23, 8.7%). One patient developed acute hemodynamic instability requiring intensive care monitoring, with resolution without permanent sequelae. One additional patient developed partial asymptomatic right portal vein branch thrombosis requiring oral anticoagulation. One major complication occurred in the control group (1/23, 4.3%): a liver abscess requiring percutaneous drainage and antibiotic therapy. All major complications were CIRSE grade 3a. No deaths occurred within 30 days in either group.

## Secondary Endpoints

### Progression-Free Survival

**Fig. 4:**
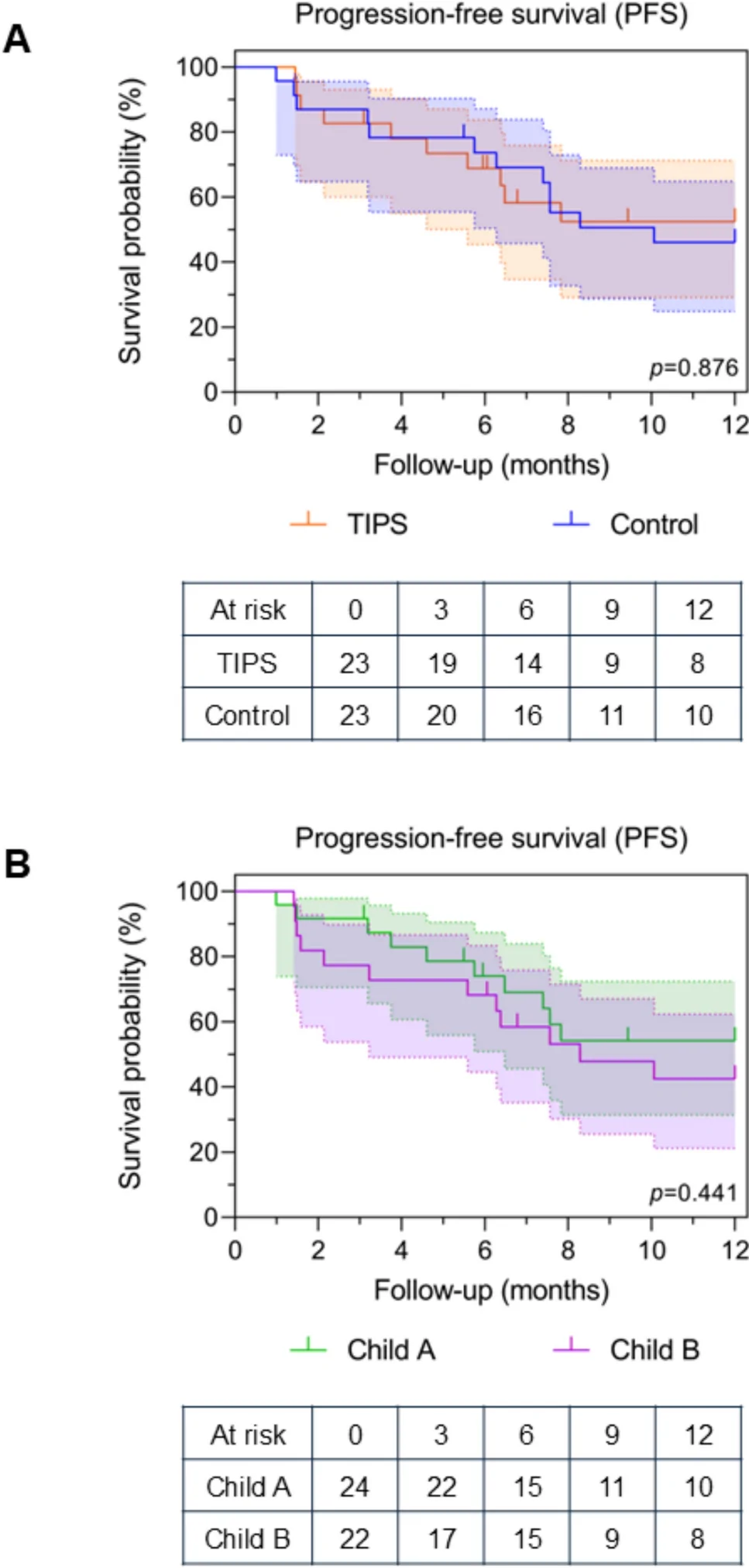
Kaplan–Meier curves for progression-free survival (PFS) over 12 months: **A** TIPS versus control group and **B** Child–Pugh A versus Child–Pugh B. Curves are shown with censoring ticks and 95% confidence bands. Numbers at risk are reported at 0, 3, 6, 9, and 12 months

For PFS, the 12-month Kaplan–Meier estimate was 52.4% in the TIPS group and 46.0% in the control group (*p* = 0.876), with 10/23 versus 12/23 events. By Child–Pugh class, 12-month PFS was 54.2% in Child–Pugh A and 42.5% in Child–Pugh B (*p* = 0.441; 10/24 vs. 12/22) (Fig. 4).

### Hepatic Decompensation-Free Survival

**Fig. 5:**
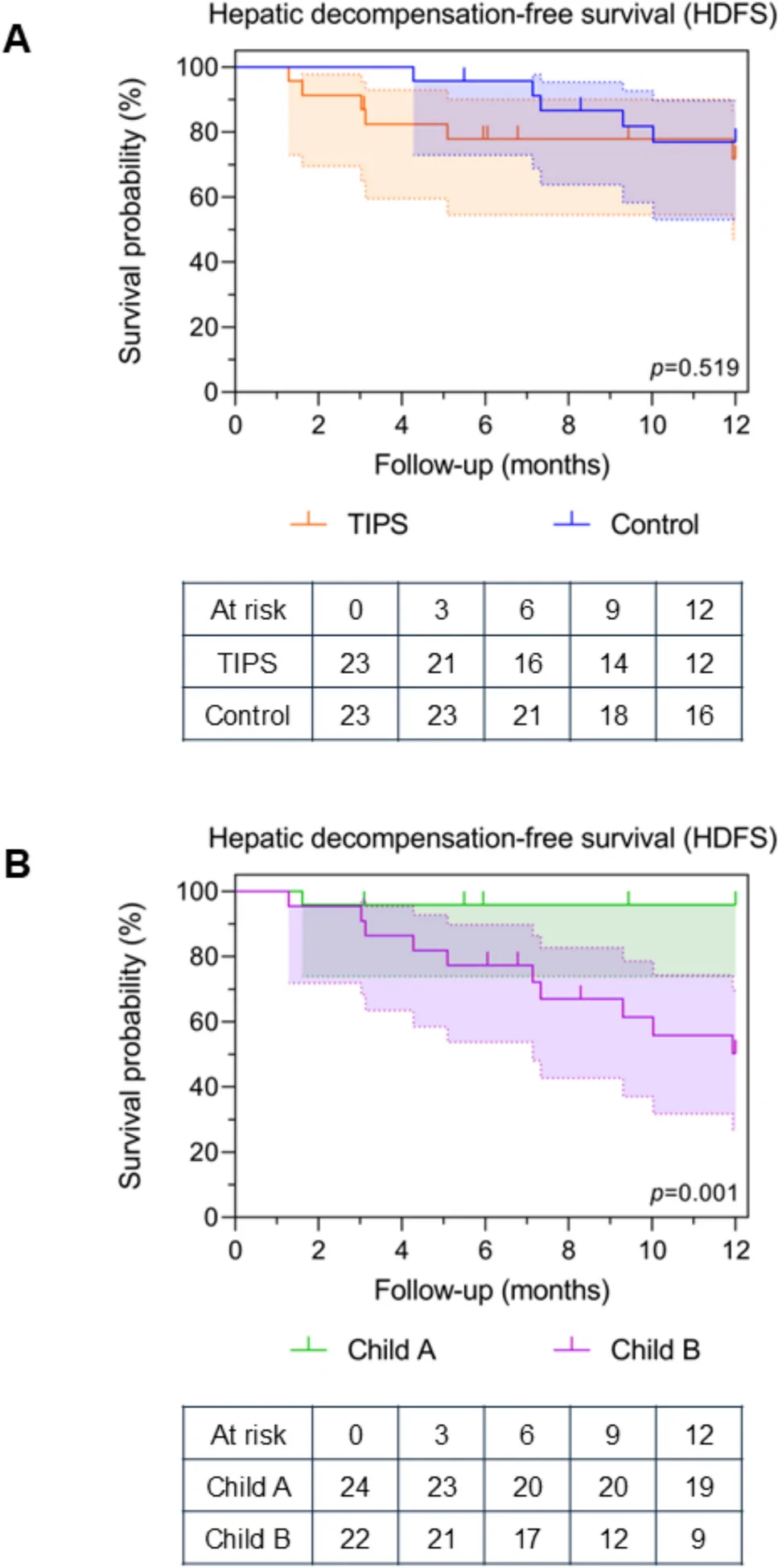
Kaplan–Meier curves for hepatic decompensation-free survival (HDFS) over 12 months: **A** TIPS versus control group and **B** Child–Pugh A versus Child–Pugh B. Curves are shown with censoring ticks and 95% confidence bands. Numbers at risk are reported at 0, 3, 6, 9, and 12 months

For HDFS, the 12-month KM estimate was 71.8% in the TIPS group and 76.9% in the control group (*p* = 0.519), with hepatic decompensation or death in 6/23 versus 5/23 patients. By Child–Pugh class, 12-month HDFS was 95.8% in Child–Pugh A and 50.2% in Child–Pugh B (*p* = 0.001; 1/24 vs. 10/22) (Fig. 5).

### Overall Survival

**Fig. 6:**
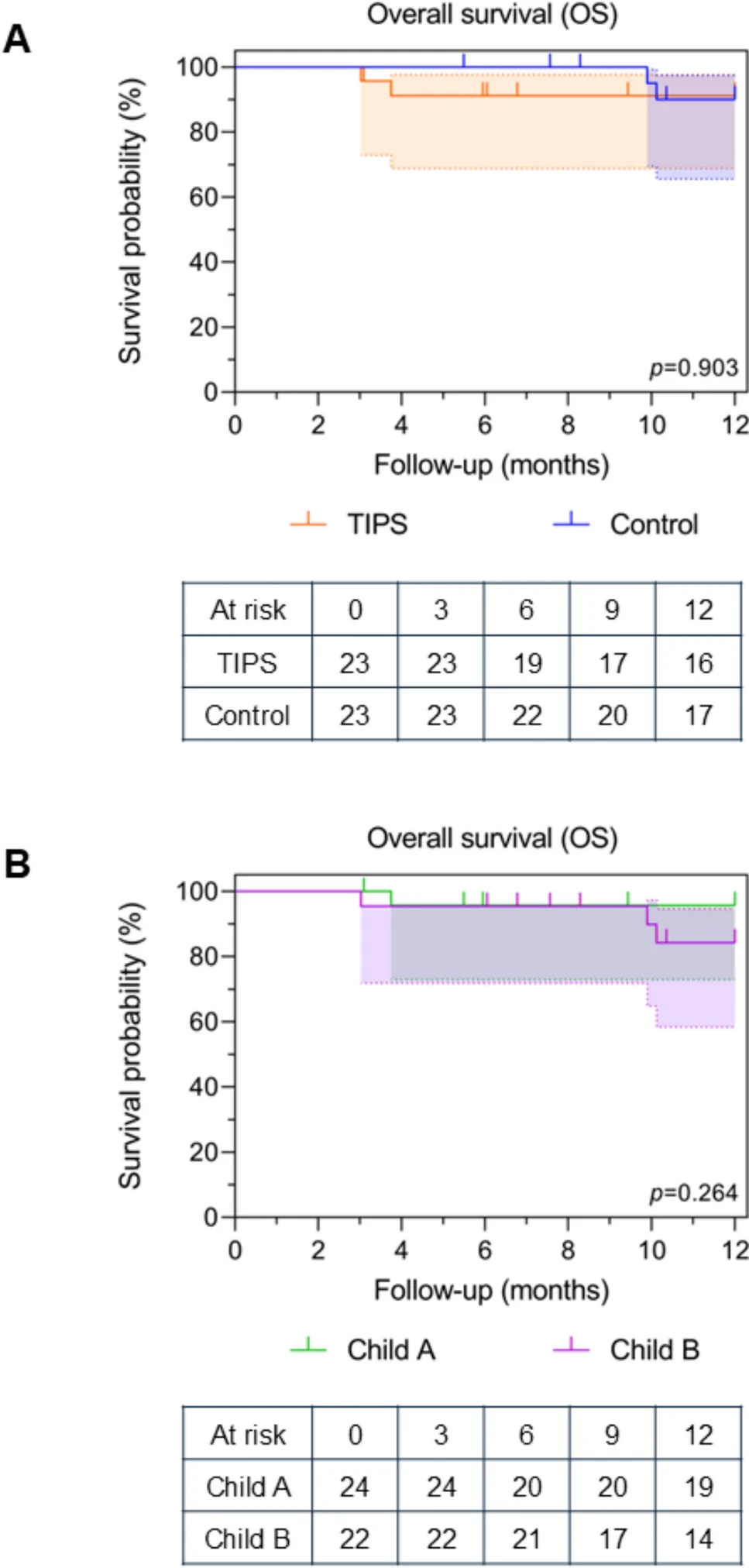
Kaplan–Meier curves for overall survival (OS) over 12 months: **A** TIPS versus control group and **B** Child–Pugh A versus Child–Pugh B. Curves are shown with censoring ticks and 95% confidence bands. Numbers at risk are reported at 0, 3, 6, 9, and 12 months

For OS, the 12-month KM estimate was 91.1% in the TIPS group and 90.0% in the control group (*p* = 0.903), with 2/23 versus 2/23 deaths. By Child–Pugh class, 12-month OS was 95.7% in Child–Pugh A and 84.2% in Child–Pugh B (*p* = 0.264; 1/24 vs. 3/22) (Fig. 6).

### Prognostic Factors

In exploratory univariable Cox regression, no variable was significantly associated with PFS or OS. Higher MELD score, MELD >  = 15, Child–Pugh B, minimum tumor-to-TIPS stent distance <  = 20 mm, and minimum ablation-zone-to-TIPS stent distance <  = 20 mm were associated with reduced HDFS (Table 3).

**Table 3:** Exploratory univariable Cox regression for key prognostic factors

Prognostic factors		PFS		OS		HDFS	
Factor	Category	HR (95% CI)	*p* value	HR (95% CI)	*p* value	HR (95% CI)	*p* value
Age (per 10 years)		1.03 (0.57–1.86)	0.924	0.46 (0.14–1.48)	0.193	0.57 (0.27–1.19)	0.136
MELD (per 1 point)		1.07 (0.94–1.23)	0.316	1.21 (0.86–1.69)	0.271	**1.33 (1.06–1.65)**	**0.012**
MELD	< 15	Reference		Reference		Reference	
	> = 15	1.26 (0.51–3.08)	0.620	0.86 (0.09–8.25)	0.895	**3.60 (1.10–11.82)**	**0.035**
Tumor size max (per 10 mm)		1.53 (0.66–3.54)	0.324	1.40 (0.20–9.72)	0.734	2.63 (0.86–8.02)	0.090
Sex	Female	Reference		Reference		Reference	
	Male	0.69 (0.23–2.06)	0.511	0.19 (0.03–1.38)	0.101	0.82 (0.18–3.80)	0.801
Child–Pugh	A	Reference		Reference		Reference	
	B	1.39 (0.60–3.21)	0.444	3.37 (0.35–32.38)	0.293	**13.46 (1.72–105.54)**	**0.013**
BCLC stage at initial HCC diagnosis	0	Reference		Reference		Reference	
	A	0.92 (0.34–2.48)	0.871	1.50 (0.14–16.52)	0.742	1.67 (0.42–6.67)	0.470
	B	2.44 (0.82–7.28)	0.110	2.04 (0.13–32.63)	0.614	1.34 (0.22–8.05)	0.746
No. of treated tumors	Solitary	Reference		Reference		Reference	
	Multiple	1.65 (0.67–4.07)	0.277	3.91 (0.54–28.13)	0.176	3.21 (0.96–10.66)	0.057
AFP positive (> 7 ng/mL)	No	Reference		Reference		Reference	
	Yes	1.85 (0.80–4.30)	0.151	1.84 (0.26–13.07)	0.543	2.39 (0.73–7.84)	0.152
Prior HCC therapy	No	Reference		Reference		Reference	
	Yes	2.04 (0.88–4.72)	0.097	0.60 (0.06–5.82)	0.663	0.36 (0.08–1.68)	0.195
Cohort	Control	Reference		Reference		Reference	
	TIPS	0.94 (0.40–2.17)	0.876	1.13 (0.16–8.03)	0.903	1.47 (0.45–4.84)	0.522
Minimum ablative margin	> = 5 mm	Reference		Reference		Reference	
	< 5 mm	1.36 (0.59–3.16)	0.470	1.53 (0.22–10.86)	0.672	0.50 (0.13–1.89)	0.308
Minimum tumor-to-TIPS stent distance	> 20 mm, TIPS only	Reference		Reference		Reference	
	< = 20 mm, TIPS only	1.80 (0.37–8.76)	0.465	Not estimable	NA	**12.86 (1.97–83.72)**	**0.008**
Minimum ablation-zone-to-TIPS stent distance	> 20 mm, TIPS only	Reference		Reference		Reference	
	< = 20 mm, TIPS only	1.17 (0.24–5.61)	0.843	Not estimable	NA	**7.80 (1.26–48.30)**	**0.027**

Analyses are exploratory due to low event counts; effect estimates may be imprecise. For TIPS proximity thresholds in Cox regression, distance was analyzed at the patient level using the minimum distance across all treated lesions. Significant results (*p* < 0.05) are shown in bold. HR, hazard ratio; CI, confidence interval; PFS, progression-free survival; OS, overall survival; HDFS, hepatic decompensation-free survival; BCLC, Barcelona Clinic Liver Cancer; AFP, alpha-fetoprotein; TIPS, transjugular intrahepatic portosystemic shunt

## Discussion

In this matched case–control study, we evaluated the safety and efficacy of stereotactic MWA for early-stage HCC in cirrhotic patients with a TIPS compared with a matched control group without TIPS. Given the limited comparative evidence in this setting [25], controls were selected using hierarchical matching based on Child–Pugh class, number of treated tumors, MELD score, tumor diameter, and ablation date, while the cohort was restricted to a uniform stereotactic technique to minimize procedural heterogeneity.

TIPS-related hemodynamic changes raise theoretical concerns about local tumor control, since increased heat sink may reduce thermal deposition and margin predictability [10–14]. In our cohorts, however, local tumor control was high, with PTE achieved in 30/31 (96.8%) lesions in both groups and only one LTP event among lesions with PTE. Quantitative margin assessment showed no significant between-group difference in minimum ablative margin or complete 5-mm margin achievement.

Early safety was also acceptable, but requires cautious interpretation. Two major complications occurred in the TIPS group and one in the control group within 30 days; no 30-day mortality was observed. The higher observed complication rate in the TIPS group was based on only one additional event, and all complications were successfully managed without permanent sequelae. The high PTE rate and absence of 30-day mortality are consistent with prior TIPS ablation series and the comparative data reported by Dumoutier et al. [22–25], and fall within pooled navigation-assisted ablation ranges reported by Tinguely et al. [27]. Taken together, these findings support the feasibility of stereotactic MWA in selected patients with early-stage HCC and TIPS, while emphasizing that the limited event numbers preclude conclusions of equivalence.

The relationship between the tumor, ablation zone, and TIPS stent remains important when interpreting these findings. Most lesions were not immediately adjacent to the shunt, although a small subset of tumors and ablation zones were within 20 mm of the TIPS stent (4/31 [12.9%] and 6/31 [19.4%], respectively). The illustrative cases in Fig. 3 highlight scenarios in which close tumor-shunt proximity may complicate margin formation or be associated with periportal vascular injury. In exploratory Cox regression, both minimum tumor-to-TIPS stent distance <  = 20 mm and minimum ablation-zone-to-TIPS stent distance <  = 20 mm were associated with reduced HDFS; however, these TIPS-only estimates were based on very small numbers and should be considered hypothesis-generating only. These findings suggest that lesions close to the TIPS stent may represent a technically and clinically distinct subgroup, but the small number of such cases precludes conclusions about a direct heat-sink effect or proximity-related hepatic risk.

Beyond local tumor control, we evaluated post-ablation outcomes in patients with TIPS, since these patients often have limited hepatic reserve and may be prone to hepatic decompensation that can affect OS and PFS [17–21]. In our study, no statistically significant between-group differences were observed in 12-month Kaplan–Meier estimates for PFS, OS, or HDFS, although the low event numbers limit inference. Liver function showed the clearest prognostic signal: Child–Pugh class stratified HDFS (12-month HDFS 95.8% in Child–Pugh A versus 50.2% in Child–Pugh B, *p* = 0.001). Exploratory Cox regression did not identify factors associated with PFS or OS. Associations with reduced HDFS were observed for hepatic reserve markers and TIPS proximity metrics; however these findings should be interpreted cautiously because of low event counts.

Clinically, the present findings suggest that stereotactic MWA may be considered in selected patients with TIPS when hepatic reserve, tumor burden, and lesion location are favorable. However, because the cohort was small and most lesions were not immediately adjacent to the shunt, these data should not be interpreted as establishing equivalent safety or efficacy across all TIPS anatomies or tumor locations. In this setting, treatment decisions should remain individualized, particularly because evidence remains limited for tumors immediately adjacent to the TIPS.

Several limitations should be acknowledged. First, this retrospective single-center study remains susceptible to selection bias and residual confounding despite matching. Second, the small cohort and low event counts, particularly for local tumor progression, limited power and precluded robust subgroup analyses. Third, follow-up was variable and analyses were restricted to 12-month endpoints. Fourth, the low number of events limited statistical power and precision; therefore, prognostic analyses were restricted to exploratory univariable Cox models, and effect estimates may be unstable and susceptible to confounding. Fifth, most tumors were not immediately adjacent to the TIPS stent, so conclusions regarding direct peri-stent heat-sink effects remain limited. Finally, generalizability may be limited because all procedures were performed using stereotactic MWA at a specialized center, with limited case numbers and event counts precluding platform-specific comparisons. Larger prospective multicenter studies with longer follow-up are needed to confirm these findings.

## Conclusion

Stereotactic MWA for early-stage HCC appears feasible in carefully selected patients with TIPS, with high local tumor control, acceptable early safety, and no 30-day mortality in this matched cohort. Larger studies are needed to better define safety, local tumor control, and liver-related outcomes, particularly for lesions close to the TIPS.

## Data Availability

The dataset is available from the corresponding author upon reasonable request.

## Abbreviations

AFP: Alpha-fetoprotein
BCLC: Barcelona clinic liver cancer
CIRSE: Cardiovascular and Interventional Radiological Society of Europe
CT: Computed tomography
HCC: Hepatocellular carcinoma
HDFS: Hepatic decompensation-free survival
IQR: Interquartile range
KM: Kaplan–Meier
LTP: Local tumor progression
MELD: Model for end-stage liver disease
MRI: Magnetic resonance imaging
MWA: Microwave ablation
OS: Overall survival
PFS: Progression-free survival
PTE: Primary technique efficacy
TACE: Transarterial chemoembolization
TIPS: Transjugular intrahepatic portosystemic shunt
